# First Molecular Evidence for Puumala Hantavirus in Poland

**DOI:** 10.3390/v6010340

**Published:** 2014-01-23

**Authors:** Hanan Sheikh Ali, Stephan Drewes, Edyta T. Sadowska, Magdalena Mikowska, Martin H. Groschup, Gerald Heckel, Pawel Koteja, Rainer G. Ulrich

**Affiliations:** 1 Friedrich-Loeffler-Institut, Federal Research Institute for Animal Health, Institute for Novel and Emerging Infectious Diseases, Südufer 10, Greifswald - Insel Riems D-17493, Germany; E-Mails: sheikh.hanan@gmail.com (H.S.A.); stephan.drewes@fli.bund.de (S.D.); martin.groschup@fli.bund.de (M.H.G.); 2 Institute of Environmental Sciences, Jagiellonian University, Kraków 30-387, Poland; E-Mails: edyta.sadowska@uj.edu.pl (E.T.S.); magdalena.mikowska@gmail.com (M.M.); pawel.koteja@uj.edu.pl (P.K.); 3 Computational and Molecular Population Genetics (CMPG), Institute of Ecology and Evolution, University of Bern, Bern CH-3012, Switzerland; E-Mail: gerald.heckel@iee.unibe.ch; 4 Swiss Institute of Bioinformatics, Genopode, Lausanne CH-1015, Switzerland

**Keywords:** *Puumala virus*, Poland, bank vole, *Clethrionomys* (*Myodes*) *glareolus*

## Abstract

*Puumala virus* (PUUV) causes mild to moderate cases of haemorrhagic fever with renal syndrome (HFRS), and is responsible for the majority of hantavirus infections of humans in Fennoscandia, Central and Western Europe. Although there are relatively many PUUV sequences available from different European countries, little is known about the presence of this virus in Poland. During population studies in 2009 a total of 45 bank voles were trapped at three sites in north-eastern Poland, namely islands on Dejguny and Dobskie Lakes and in a forest near Mikołajki. S and M segment-specific RT-PCR assays detected PUUV RNA in three animals from the Mikołajki site. The obtained partial S and M segment sequences demonstrated the highest similarity to the corresponding segments of a PUUV strain from Latvia. Analysis of chest cavity fluid samples by IgG ELISA using a yeast-expressed PUUV nucleocapsid protein resulted in the detection of two seropositive samples, both being also RT-PCR positive. Interestingly, at the trapping site in Mikołajki PUUV-positive bank voles belong to the Carpathian and Eastern genetic lineages within this species. In conclusion, we herein present the first molecular evidence for PUUV in the rodent reservoir from Poland.

## 1. Introduction

Hantaviruses, family *Bunyaviridae*, are enveloped viruses with a trisegmented RNA genome of negative polarity [1]. These zoonotic viruses have been initially believed to be exclusively rodent-borne. However, in recent years a large number of novel hantaviruses has been identified in shrews, moles and bats [2]. Human disease seems to be caused exclusively by certain rodent-borne hantaviruses. Their transmission to humans is mediated via inhalation of aerosolized excreta from persistently infected rodent hosts. Following the distribution of the causative hantaviruses, two different clinical syndromes have been described in the Old World and New World. The Hantavirus Cardiopulmonary Syndrome (HCPS) in the Americas is characterized by a very high case fatality rate of up to 35%, whereas the case fatality rate of the Haemorrhagic Fever with Renal Syndrome (HFRS) in Europe and Asia ranges from 0.1% to 15% [3,4].

In Europe, hantavirus infections are, for a long time, found in Fennoscandia, Russia and the Balkan region. Currently, hantavirus infections have been detected with highly variable annual case numbers in human patients and were identified in different reservoir species in most of Europe [5]. In certain parts of Europe hantavirus infections are frequently reported, e.g., the central and eastern parts of Finland, the northern part of Sweden, the Ardennes region in Belgium and France, the southern, western and northwestern part of Germany, the Balkans and certain regions of European Russia. In addition, for some parts of Europe, e.g., Baltic countries, Hungary and Greece, a high seroprevalence was contrasted by a low number of reported HFRS cases. For further countries, e.g., UK and Ukraine, no comprehensive epidemiological data are available [6].

*Puumala virus* (PUUV) causes the vast majority of human hantavirus infections in Fennoscandia, Central and Western Europe. Although severe and even a few fatal human cases have been reported, PUUV causes usually in humans a mild to moderate form of HFRS called nephropathia epidemica with a very low case fatality rate [7,8,9]. The bank vole *Clethrionomys glareolus* (*Myodes glareolus*) [10] represents the reservoir of this hantavirus [11]. Closely related viruses have been identified in other *Clethrionomys* species in Asia [12,13]. Consistent with the distribution of the bank vole in the main parts of continental Europe PUUV was detected in most European countries including Finland, north Sweden, Norway, Germany, Belgium, Luxembourg, France, the Netherlands, Austria, Slovakia, Latvia, Estonia, Lithuania, Slovenia, Croatia, Bosnia and Hercegovina, Greece and the European part of Russia [7,14,15,16,17,18,19,20,21,22,23,24,25,26,27,28,29,30]. An oscillation of the recorded number of human cases in previous years has been reported for Belgium, France and Germany, and was postulated to be caused by a mass reproduction of the bank vole following beech mast years [31].

Little is known about the presence of human pathogenic hantaviruses in Poland, although the reservoirs of PUUV (bank vole) and *Dobrava-Belgrade virus*, genotype Kurkino (striped field mouse *Apodemus agrarius*) are present in large areas of the country [32]. In Poland, hantavirus-caused infections are notifiable under the national epidemiological surveillance system since 2007, but the number of officially recorded cases remains low: 3–9 cases per year (which includes also cases related to hantaviruses other than PUUV) [33]. In addition, human cases were exclusively identified by serological investigations without molecular characterization [34,35,36]. A serologic survey among mammalogists revealed higher reactivity with PUUV antigen than with *Hantaan virus* (HTNV) antigen indicating that these persons had rather contact with PUUV or the related *Tula virus* (TULV) than with HTNV-related DOBV [37]. In addition, hantavirus-specific antibodies have been detected in a high-risk group such as forest workers in north-eastern Poland [38]. In contrast, few molecular studies in reservoirs confirmed the presence of hantaviruses in Poland. TULV strain Lodz was isolated in cell culture from tissue samples of the common vole *Microtus arvalis* trapped in a region 53 km south and 104 km west of Warsaw, where human and animal hantavirus infections were not reported [39]. In addition, a molecular survey of 60 small mammals in eastern Poland revealed the presence of hantaviruses in four species: the prevalence was in *A. agrarius* (*n* = 39) 2.6%, and in *Microtus agrestis* (*n* = 1), *C. glareolus* (*n* = 5), *Sorex araneus* (*n* = 8) 12.5%–100%. The L segment sequence analysis of 5 samples revealed nucleotide sequence identities of 77%–86% to hantavirus sequences of Fusong-Mf-731 detected in *Microtus fortis* from China [40]. Recently, a novel shrew-borne hantavirus (Boginia virus) was identified in the Eurasian water shrew *Neomys fodiens* in central Poland [41].

In this study bank voles trapped in north-eastern Poland were investigated by serology and RT-PCR for the presence of PUUV infections.

## 2. Results and Discussion

**Figure 1:**
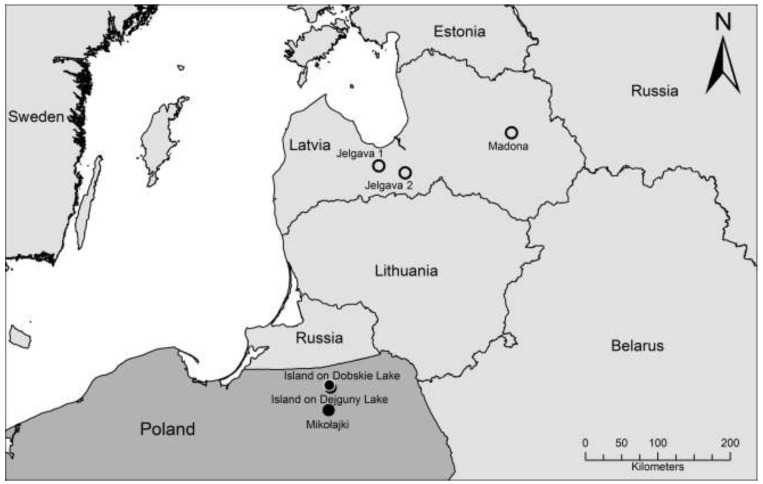
Map of the north-eastern part of Poland and the surrounding countries showing the three trapping sites of bank voles in Poland described here and the three localities in Latvia where related PUUV sequences were detected [42].

During August 2009 a total of 45 bank voles were trapped in the north-eastern part of Poland. The trapping sites were located in a forest close to Mikołajki with a distance of *ca.* 30 and 35 km, respectively, to the other two trapping sites on islands on Dejguny and Dobskie Lakes (Figure 1). Serological screening of chest cavity fluid from 44 of 45 bank voles by an in-house IgG enzyme-linked immunosorbent assay (ELISA) using a yeast-expressed PUUV nucleocapsid protein revealed two seropositive samples, both from animals trapped in Mikołajki forest (Table 1).

**Table 1:** Results of the serological and S segment RT-PCR investigations of bank voles trapped at three sites in Poland.

Trapping site	Number of positive/total number of investigated animals	
	Serology	S-RT-PCR
Mikołajki forest	2/15 *	3/16
Dobskie island	0/16	0/16
Dejguny island	0/13	0/13

* from a single animal no chest cavity fluid for serology was available.

**Figure 2:**
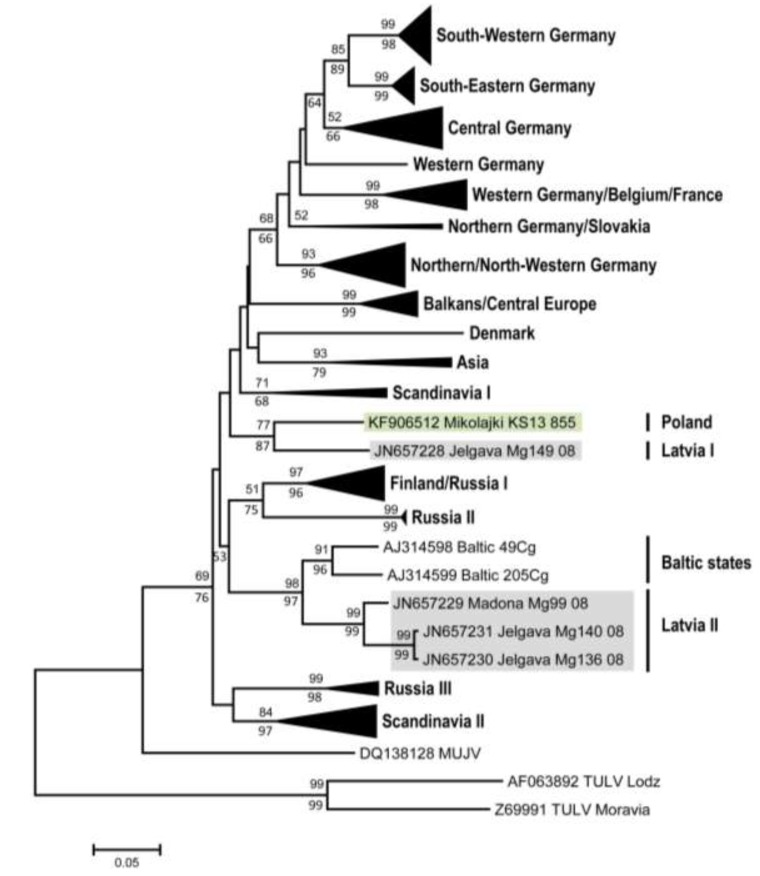
Phylogenetic relationships of *Puumala virus* sequences based on 465 nt of the S segment spanning positions 436–900 (numbering based on strain Sotkamo, accession number NC_005224) with *Tula virus* strains Lodz AF063892 and Moravia Z69991 as outgroup. The identical novel S segment sequence from the three bank voles (KS13/855, KS13/856 and KS13/861) in Mikołajki (Poland) and relevant sequences from Latvia (see Figure 1) are highlighted. Other geographical clusters of closely related sequences were condensed to triangles with sizes proportional to sequence numbers. Support values for Neighbor-Joining or Bayesian phylogenetic analyses are reported above/below branches of main nodes if they exceeded 50 percent.

Subsequent screening of lung tissue by PUUV/TULV S segment RT-PCR test revealed three positive samples from male bank voles (with two being seropositive and one being seronegative) from the forest near Mikołajki (Table 1). Cloning of the amplification products and sequencing confirmed identical sequences of 711 nucleotides length for all three samples (accession number KF906512). A BLASTn search identified the sequence as a PUUV sequence with the highest similarity to a PUUV sequence derived from a bank vole originated from Jelgava, Latvia (Mg149/2008; JN657228; Jelgava 1, Figure 1). A phylogenetic analysis of this novel S segment sequence confirmed its closest similarity to this sequence from Jelgava 1 and showed clear a distinctness from other PUUV sequences in the Baltic region, Russia or Germany (Figure 2). The nucleotide sequence divergence of the novel sequence from Poland to the sequence from Jelgava 1 was 11.2%, whereas the sequence divergence to other two sequences from Latvia ranged from 17.5% to 19.2% (Table 2). The amino acid sequences of the novel strain and that from Jelgava 1 were identical, but the corresponding amino acid sequences from the other sites in Latvia varied by 4.6% to 5.2%. The nucleotide sequence divergence of the Mikołajki sequence to representative sequences covering the distributional range of PUUV in Europe reached 14.0% to 21.3%.

**Table 2:** Nucleotide (above the diagonal) and amino acid sequence identity of the novel S segment and nucleocapsid protein sequence from Mikołajki with corresponding sequences from Latvia, Croatia, Finland, Sweden, Germany and Denmark.

PUUV strains	Mikołajki	Jelgava 1	Jelgava 2	Madona	Gerovo	Konnevesi	Vindeln	Bavaria	Fyn
	KS13 855	Mg149 08	Mg136 08	Mg99 08	Mg979 08	Mg M114B 05	L20Cg 83	Mu CG 9 04	19
Mikołajki KS13 855		0.888	0.808	0.825	0.827	0.860	0.819	0.819	0.787
Jelgava 1 Mg149 08	1		0.832	0.823	0.817	0.843	0.832	0.819	0.817
Jelgava 2 Mg136 08	0.948	0.948		0.948	0.789	0.806	0.806	0.780	0.782
Madona Mg99 08	0.954	0.954	0.980		0.780	0.812	0.812	0.797	0.776
Gerovo Mg979 08	0.954	0.954	0.922	0.935		0.821	0.810	0.825	0.800
Konnevesi Mg M114B 05	0.967	0.967	0.941	0.961	0.948		0.832	0.815	0.787
Vindeln L20Cg 83	0.967	0.967	0.935	0.954	0.935	0.948		0.817	0.819
Bavaria Mu CG 9 04	0.974	0.974	0.935	0.948	0.961	0.961	0.948		0.791
Fyn 19	0.961	0.961	0.922	0.935	0.935	0.935	0.941	0.954	

For two of the three S segment positive lung tissue samples a M segment RT-PCR amplification product of the expected size was obtained. Interestingly, both samples originated from the seropositive animals (KS13/855; KS13/856). The M segment sequences from both samples were found to be identical (accession number KF906513; length 618 nucleotides). Pairwise comparisons of a 177 nt-long segment of this sequence showed a 82.4% and 83% sequence identity to sequences from Latvia (Jelgava 1/Mg 149; JN657233) and Croatia (Gerovo Mg982; KC676635), respectively (data not shown). The corresponding amino acid sequence of 59 residues length was found to be identical to sequences from Jelgava 1 and Bavaria or showed a divergence of 5.1%–11.9% to other European PUUV strains (data not shown).

**Figure 3:**
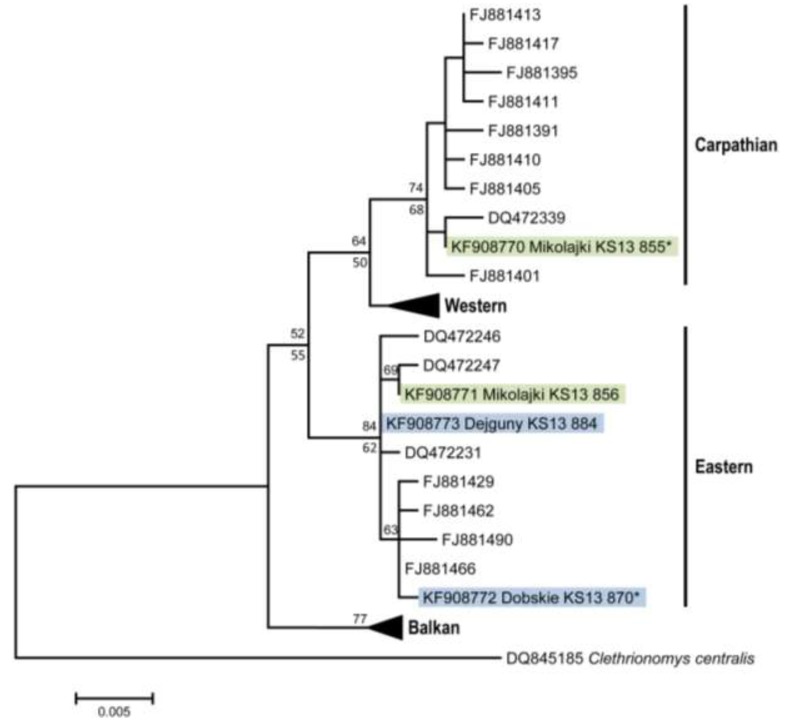
Phylogenetic relationships of *cytochrome b* sequences (843 nt) in *Clethrionomys glareolus* from the study region (highlighted) relative to major phylogeographic lineages (Carpathian, Western, Eastern, Balkan; [43]) within the species. Representative sequences from all phylogeographic lineages present in the larger region (see [43]) were obtained from GenBank, and *Clethrionomys* (*Myodes*) *centralis* was used as outgroup. Clusters of phylogeographic lineages without novel sequences from Poland in this study were condensed to triangles with sizes proportional to sequence numbers. Support values for Neighbor-Joining or Bayesian phylogenetic algorithms exceeding 50 percent are reported above/below main branches. * Identical sequences: KS13/855 = KS13/861; KS13/870 = KS13/868.

Phylogenetic analysis of *cytochrome b* sequences from the three PUUV-positive bank voles from Mikołajki forest and three animals from the island populations revealed the presence of representatives of the Carpathian and Eastern genetic lineages within the species (Figure 3). Interestingly, two of three PUUV-RNA-positive animals from the forest population belong to the Carpathian lineage, whereas the third one belongs to the Eastern lineage. The detection of both bank vole lineages in this region is in line with previous reports of a contact zone between these lineages in Poland [43]. The animals from the island populations belong to the Eastern lineage.

## 3. Experimental Section

### 3.1. Rodent Trapping and Dissection

Bank voles were trapped in August 2009 in Mazurian Lake Range (north-eastern Poland) at three sites (Figure 1): an island on Dejguny Lake (54°2'N, 21°37'E, area 9.3 ha, about 250 m from mainland), an island on Dobskie Lake (54°5'N, 21°36'E) and a forest near Mikołajki (53°46'N, 21°30'E). In each of the sites, live-traps were set for 3–5 days and were checked every morning. The trapped individuals were maintained temporarily in standard plastic mouse cages in groups of up to four same-sex individuals from the same population and then transported to the Institute of Environmental Sciences (Jagiellonian University, Kraków, Poland). Immediately after arrival in the laboratory (within 1–12 days after trapping), the animals were killed by decapitation and dissected. Kidney, liver and parts of auricle were stored for toxicological and molecular analyses and the remaining carcasses were stored at −75 °C. All the procedures were carried out according to EC Directive 86/609/EEC for animal experiments and were approved by the First Local Bioethical Committee in Kraków (decision # 48/2007). For the purpose of this study, 45 frozen carcasses were transferred in dry ice to the Friedrich-Loeffler-Institut (Greifswald - Insel Riems, Germany): 7 males and 6 females from Dejguny, 7 males and 9 females from Dobskie and 9 males and 7 females from Mikołajki. The lung tissue and tail samples of these 45 carcasses were collected according to a standard protocol; chest cavity fluid was obtained by addition of 1 mL sterile phosphate-buffered saline (PBS).

### 3.2. Serological Investigations

The chest cavity fluid of dissected bank voles was screened by an IgG ELISA based on a yeast expressed-nucleocapsid protein of PUUV strain Bavaria [44]. The ELISA was performed according to a previously established protocol [45].

### 3.3. Nucleic Acid Isolation, Hantavirus RT-PCR, *cytochrome b* PCR and Sequence Determination

RNA was extracted from lung tissue samples of bank voles using Qiazol solution (Qiagen, Hilden, Germany). The RT-PCR amplification follows previously published protocols for amplification of partial S and M segment sequences [44,45]. For amplification 2.5 µL of RNA using 10 pmol of primers (S segment: 342 forward, 1102 reverse; M segment: C1, C2) and SuperScript III one step RT-PCR kit (Invitrogen, Darmstadt, Germany) in a final volume of 25 µL were applied under the following amplification conditions: reverse transcription at 50 °C for 45 min, inactivation of reverse transcriptase at 94 °C for 2 min, 40 cycles of denaturation at 94 °C for 30 s, annealing at 46 °C (for S segment) and at 58.5 °C (for M segment) for 30 s, and elongation at 68 °C for 1 min, and final extension at 68 °C for 10 min.

Amplification products were run in agarose gels and visualized by UV illumination after ethidium bromide staining. RT-PCR products of expected size were cloned into vector pCR2.1-TOPO^®^ (TopoTA cloning kit, Invitrogen). The plasmids were purified using QIAprep spin Miniprep kit according to the manufacturer’s instructions (Qiagen, Hilden, Germany).

For *cytochrome b* PCR analysis, tail tip samples of 0.5 mm from selected animals were lysed over night at 56 °C and 400 rpm in 300 µL lysis buffer (50 mM KCl, 10 mM Tris-HCl (pH 9.0), 0.45% Nonidet P40, 0.45% Tween 20) containing 3 µL proteinase K (10 mg/mL). The *cytochrome b*-specific PCR was performed as described previously [46].

The plasmid DNAs and *cytochrome b* PCR products were sequenced using the BigDye^®^ Terminator v1.1 Cycle Sequencing Kit (Perkin-Elmer, Waltham, MA, USA) on an ABI 310 Genetic Analyser (Applied Biosystems, Foster City, CA, USA). To prove a potential quasispecies structure 13, 13 and 18 S segment plasmids of the animals KS13/855, KS13/856 and KS13/861, respectively, and 14 and 4 M segment plasmids of KS13/855 and KS13/856, respectively, were sequenced.

### 3.4. Sequence Comparison and Phylogenetic Analyses

An initial comparison of the novel PUUV sequences with existing data was done using the BLAST search module [47].

For phylogenetic analyses of PUUV and *cytochrome b* sequences, published sequences were included in the analyses in order to cover the known genetic diversity and geographical distribution of PUUV and bank voles widely, which is analogous to previous investigations [44,48,49]. For the PUUV S segment, the 465 nt alignment contained 165 sequences including four sequences from Latvia (Madona Mg99, Jelgava Mg136, Jelgava Mg140, Jelgava Mg149 [42]), and sequences of TULV strains Lodz AF063892 and Moravia Z69991 as outgroup. For the M segment, the analyses covered 177 nt with a similar geographical spread of sequences and the same outgroup strains. For the identification of the genetic lineage of the bank voles, *cytochrome b* sequences of the lineages Eastern, Carpathian, Balkan and Western, which have been found previously in the larger region [43], were retrieved from GenBank. Phylogenetic analyses involved the estimation of the optimal nucleotide substitution model based on the Bayesian information criterion in Mega version 5.2 [50] before tree reconstruction with Neighbor-Joining (NJ) algorithms incorporated in Mega and Bayesian algorithms incorporated in MrBayes 3.1.2. [51]. For analyses of the PUUV S segment, the GTR model with gamma shape parameter and invariable sites was used with the estimated values, and for the *cytochrome b* sequences (843 nt) the T3P model with gamma parameter and a fraction of invariable sites was applied. For the NJ analyses, 5,000 bootstrap replicates were performed for each data set. Bayesian analyses were run two times for each dataset with one cold and three hot chains for 5 million generations with every 10th generation sampled. The first 25% of the samples were discarded as burn-in and convergence of chains was confirmed according to standard procedures (see [52] for details).

## 4. Conclusions

This study shows first molecular evidence of PUUV in the north-eastern part of Poland. The finding of PUUV-positive bank voles of the Carpathian and Eastern genetic lineage demonstrates that both genetic lineages, as also the Western lineage in Germany, Belgium and France [43,44], are susceptible to PUUV infection. In conclusion, the findings have implications for the awareness of the physicians in Poland and public health measures in north-eastern Poland. Future studies will have to test for potential associations of the PUUV prevalence and bank vole population dynamics, examine reasons for the low prevalence of PUUV in bank voles from north-eastern Poland and the influence on the frequency of human infections in this part of Poland.
